# eGFR slope as a surrogate endpoint for end-stage kidney disease in patients with diabetes and eGFR > 30 mL/min/1.73 m^2^ in the J-DREAMS cohort

**DOI:** 10.1007/s10157-023-02408-z

**Published:** 2023-10-09

**Authors:** Yuka Sugawara, Eiichiro Kanda, Mitsuru Ohsugi, Kohjiro Ueki, Naoki Kashihara, Masaomi Nangaku

**Affiliations:** 1 Division of Nephrology and Endocrinology, The University of Tokyo, Tokyo, Japan; 2 Medical Science, Kawasaki Medical School, Okayama, Japan; 3 Department of Nephrology and Hypertension, Kawasaki Medical School, Okayama, Japan; 4 Department of Diabetes, Endocrinology and Metabolism, National Center for Global Health and Medicine, Tokyo, Japan; 5 Diabetes and Metabolism Information Center, National Center for Global Health and Medicine, Tokyo, Japan; 6 Diabetes Research Center, National Center for Global Health and Medicine, Tokyo, Japan

**Keywords:** eGFR slope, Diabetic kidney disease, Chronic kidney disease, Surrogate endpoint

## Abstract

**Background:**

An analysis of European and American individuals revealed that a reduction in estimated glomerular filtration rate (eGFR) slope by 0.5 to 1.0 mL/min/1.73 m^2^ per year is a surrogate endpoint for end-stage kidney disease (ESKD) in patients with early chronic kidney disease. However, it remains unclear whether this can be extrapolated to Japanese patients.

**Methods:**

Using data from the Japan diabetes comprehensive database project based on an advanced electronic medical record system (J-DREAMS) cohort of 51,483 Japanese patients with diabetes and a baseline eGFR ≥ 30 mL/min/1.73 m^2^, we examined whether the eGFR slope could be a surrogate indicator for ESKD. The eGFR slope was calculated at 1, 2, and 3 years, and the relationship between each eGFR slope and ESKD risk was estimated using a Cox proportional hazards model to obtain adjusted hazard ratios (aHRs).

**Results:**

Slower eGFR decline by 0.75 mL/min/1.73 m^2^/year reduction in 1-, 2-, and 3-year slopes was associated with lower risk of ESKD (aHR 0.93 (95% confidence interval (CI) 0.92–0.95), 0.84 (95% CI 0.82–0.86), and 0.77 (95% CI 0.73–0.82), respectively); this relationship became more apparent as the slope calculation period increased. Similar results were obtained in subgroup analyses divided by baseline eGFR or baseline urine albumin-creatinine ratio (UACR), with a stronger correlation with ESKD in the baseline eGFR < 60 mL/min/1.73 m^2^ group and in the baseline UACR < 30 mg/gCre group.

**Conclusion:**

We found that changes in the eGFR slope were associated with ESKD risk in this population.

**Supplementary Information:**

The online version contains supplementary material available at 10.1007/s10157-023-02408-z.

## Introduction

Chronic kidney disease (CKD) is a syndrome characterized by a gradual decline in kidney function, affecting approximately 10–15% of the world's population and 13% of the Japanese population [1–3]. In end-stage kidney disease (ESKD), renal function becomes obsolete, and patients require kidney replacement therapy, such as hemodialysis. Patients with ESKD have increased mortality and cardiovascular events rates [4], and kidney replacement therapy reduces their quality of life despite being necessary [5]. Furthermore, hemodialysis is also economically burdensome due to its high medical expenditures [6]. Thus, there is a pressing need to develop measures to prevent CKD progression to ESKD.

Few medicines can slow CKD progression. In Japan, only SGLT2 inhibitors have been approved for CKD without specific restrictions. Renin–angiotensin–aldosterone system (RAAS) inhibitors are also administered for kidney protection; however, in Japan, they can be administered for hypertension, not for CKD. In nephrology, the number of randomized clinical trials is fewer than those in the other fields [7] because of the difficulty in designing appropriate clinical trials and determining appropriate endpoints. This may be a hurdle for the approval of new drugs. To address this, a 30–40% drop in the estimated glomerular filtration rate (eGFR) was proposed as a surrogate endpoint for ESKD at an international workshop organized by the National Kidney Foundation (NKF) and Food and Drug Administration in 2014 [8]. The "Guidelines for clinical evaluation of chronic kidney disease" for Japanese patients with CKD were established in 2018 after this surrogate endpoint was confirmed to be applicable in Japanese patients [9]. However, these guidelines are applicable for relatively late-stage CKD, and the surrogate endpoint is not considered suitable for early-stage CKD. In 2019, an eGFR slope reduction of 0.5 to 1.0 ml/min/1.73 m^2^ was proposed as a surrogate endpoint for early-stage CKD at an international workshop hosted by NKF [10]. However, the data did not include the Japanese population. Therefore, it must be verified whether this surrogate endpoint may be used in Japan.

Causes of CKD include a wide variety of kidney diseases, including diabetic kidney disease (DKD), which accounts for many CKD cases in Japan. Herein, we used data from the “Japan Diabetes comprehensive database project based on an advanced electronic medical record system (J-DREAMS)” [11], a representative cohort of diabetes cases in Japan, to determine whether eGFR slope could be a surrogate endpoint for ESKD.

## Materials and methods

### Database

The details of J-DREAMS are elsewhere [11]. J-DREAMS developed a database of patients with diabetes to understand their treatment and improve it. This study has registered 79,000 patients with diabetes in Japan since 2014, and registrations are still being accepted. Doctors at participating facilities fill out a database template for each patient’s medical record. Data on the patient's physical condition, life history, and complications were collected. Additionally, laboratory values and medication data for 3 months before the examination were also collected.

All procedures were performed in accordance with the ethical standards of the Research Committee of the National Center for Global Health and Medicine (No: NCGM-G-002354-00) and with those of the 1964 Helsinki Declaration and its later amendments. According to the Guidelines for Epidemiological Studies of the Ministry of Health, Labor, and Welfare of Japan, written informed consent was not required due to the retrospective nature of the study.

### Subject group

Using data from 51,483 J-DREAMS-registered patients with eGFR ≥ 30 mL/min/1.73 m^2^, the following analyses were performed. Patients who developed ESKD during the eGFR slope calculation period were excluded from this analysis. Patients without data after the calculation period were excluded. Patients with baseline eGFR of > 200 mL/min/1.73 m^2^ were excluded.

### Observation period for events

**Fig. 1:**
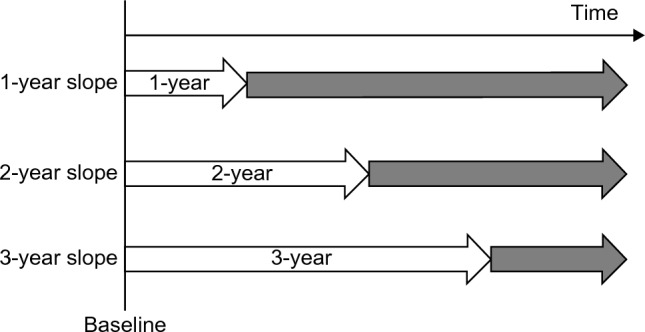
Estimated glomerular filtration rate (eGFR) slope calculation and observation periods for events. The eGFR value at the beginning of the observation period was used as the baseline value. The 1-year eGFR slope was calculated for cases with eGFR records at two time points: baseline and 1 year ± 3 months from baseline. In the same way, 2- and 3-year eGFR slopes were calculated. The period from baseline until the date when the end-stage kidney disease event occurred or the last observation date was defined as the observation period for events (grey arrows), excluding the period used for the slope calculation (white arrows)

The baseline of the observation period was defined as the date of first reported eGFR of ≥ 30 mL/min/1.73 m^2^ after individual enrollment in the J-DREAMS in 2014 or later. The observation period ended when the ESKD event, the last eGFR, or the last template entry occurred until August 27, 2020. This analysis used data from this period. The eGFR slope calculation period was excluded from the observation period for events (Fig. 1**).** A pure ESKD event included the initiation of kidney replacement therapy, while a composite ESKD event included eGFR < 15 mL/min/1.73 m^2^ and the initiation of kidney replacement therapy. The risks for these two events were analyzed.

### Definition of eGFR slope

The eGFR was calculated using serum creatinine levels measured for clinical purposes at each participating center using the Japanese formula [12]. The eGFR slope was calculated using a linear mixed-effects (ME) model, as described in the previous paper [13]. For comparison, we also calculated the slope using an ordinary least-squares linear regression (OLS) model. When calculating slope, we considered eGFR data at the beginning of the observation period as baseline (year 0), and the eGFR slope for 1-year (1-year slope) was calculated for cases with eGFR records at the following two time points: (1) baseline and (2) 1 year ± 3 months from the baseline. All eGFR records during this period were used to calculate the eGFR slope. The same method was used for 2 or 3 years (2-year and 3-year slopes, respectively).

### Covariates

Age, sex, smoking status, history of cardiovascular disease, administration of RAAS inhibitors, baseline eGFR value, and the natural logarithm of the baseline urine albumin-to-creatinine ratio (UACR) were clinically significant factors for this analysis. A history of cardiovascular disease was defined as a history of coronary artery disease, heart failure, stroke, peripheral artery disease, or lower-extremity amputation. The baseline UACR had been measured for medical purposes at each J-DREAMS-participating center.

### Multiple imputations

Among the covariates, only the baseline UACR was missing in some cases. Assuming that this data was “missing at random,” they were replaced using the multiple imputation by chained equations algorithm [14]. In particular, the aregImpute of R was used. The ESKD outcome, follow-up period, and covariates to be used in the subsequent Cox proportional hazards model were entered into the algorithm, and the number of iterations was set to 20.

### Statistical analysis

The baseline basic and clinical characteristics were classified into four subgroups according to the 2-year slope value (< −5, −5 to −2.5, −2.5 to 0, ≥ 0 mL/min/1.73 m^2^/year). For the main analysis, the relationships between the 1-, 2-, and 3-year slopes and pure ESKD risk or composite ESKD risk were estimated using Cox proportional hazards models for adjusted hazard ratios (aHR) after multiple imputations. The composite ESKD risk was considered the primary outcome of interest. Subgroup analyses were also performed by dividing the cohort based on the baseline eGFR values (G1 and G2: eGFR ≥ 60 mL/min/1.73 m^2^; G3a and G3b: eGFR 30–60 mL/min/1.73 m^2^), or the baseline UACR (A1: UACR < 30 mg/Cre; A2 and A3: UACR ≥ 30 mg/gCre).

Age, sex, cardiovascular disease history, smoking history, RAAS inhibitors administration, baseline eGFR, and natural logarithm of the baseline UACR were adjusted. The following covariates for which the Cox proportional hazards model did not converge were excluded from stratified analysis by albuminuria: smoking status and history of cardiovascular disease.

All statistical analysis was performed using R statistical Software (version 4.3.1; R Foundation for Statistical Computing, Vienna, Austria). The association was considered statistically significant as defined by a 95% confidence interval (95% CI) of aHR that does not cross 1.

## Results

### Baseline participant characteristics

Among the 51,483 J-DREAMS registrants with eGFR values ≥ 30 mL/min/1.73 m^2^, the 1-, 2-, and 3-year eGFR slopes could be calculated for 16,078, 12,435, and 8334 participants when analyzing a composite ESKD risk, respectively. Medians of 8 (interquartile range (IQR) 6–11), 11 (IQR 7–16), and 14 (10–21) eGFR recording were used for calculation of the 1-, 2-, and 3-year slopes. The mean observation period for events was 463 days (standard deviation [SD] ± 335 days), 419 days (SD ± 250 days), and 215 days (SD ± 125 days) for the 1-, 2-, and 3-year slopes, respectively (Fig. 1). Among the adjustment factors, only the baseline UACR values were missing in some; therefore, multiple imputations were performed. The number of cases with missing baseline UACR values was 9,095 (56.6%), 7,533 (60.6%), and 5,598 (67.2%) for the 1-, 2-, and 3-year slopes. Of the eligible patients with 1-, 2-, and 3-year slopes, 79 (0.49%), 83 (0.67%), and 32 (0.38%) developed composite ESKD events. Overall, the mean age was 63.7 years (SD ± 13.5 years), 57% of the patients were male, the mean baseline eGFR was 72.2 mL/min/1.73 m^2^ (SD ± 21.9 mL/min/1.73 m^2^), the median baseline UACR was 20.0 mg/gCre (interquartile range 9.5–74.0 mg/gCre), and the mean eGFR slope was -1.8 mL/min/1.73 m^2^/year (SD ± 3.3 mL/min/1.73 m^2^/year) (Table 1).

**Table 1:** Basic and clinical characteristics of participants based on the 2-year eGFR slope calculated under mixed-effects model

	Total	2-year slope			
		slope < −5 mL/min/1.73 m^2^ /year	−5 ≤ slope < −2.5 mL/min/1.73 m^2^ /year	−2.5 ≤ slope < 0 mL/min/1.73 m^2^ /year	slope ≥ 0 mL/min/1.73 m^2^ /year
	N = 12,435	N = 1253	N = 2822	N = 5474	N = 2886
Age, years, mean (SD)	63.7 (13.5)	58.9 (15.3)	63.5 (13.4)	65.3 (12.5)	62.8 (13.8)
Male, n (%)	7088 (57%)	662 (53%)	1550 (55%)	3259 (60%)	1617 (56%)
Type-1 diabetes mellitus, n (%)	1107 (9%)	126 (10%)	255 (9.0%)	459 (8.4%)	267 (9.3%)
Smoking status					
Current smoker, n (%)	1430 (11%)	198 (15%)	354 (13%)	538 (10%)	340 (12%)
Past smoker, n (%)	2489 (20%)	264 (21%)	556 (20%)	1079 (20%)	590 (20%)
Body mass index, kg/m^2,^ mean (SD)	24.8 (4.3)	24.7 (4.6)	24.8 (4.2)	24.7 (3.9)	25.2 (5.3)
Baseline eGFR, mL/min/1.73m^2^, mean (SD)	72.2 (21.9)	88.3 (28.2)	74.9 (21.3)	67.9 (18.5)	71.0 (21.8)
CKD stage					
G1, n (%)	2162 (17%)	543 (43%)	578 (21%)	595 (11%)	446 (16%)
G2, n (%)	6623 (53%)	531 (42%)	1571 (56%)	2991 (55%)	1530 (53%)
G3a, n (%)	2555 (21%)	125 (10%)	472 (17%)	1323 (24%)	635 (22%)
G3b, n (%)	1095 (9%)	54 (4%)	201 (7%)	565 (10%)	275 (10%)
eGFR slope, mL/min/1.73m^2^ /year, mean (SD)	− 1.8 (3.3)	− 8.2 (4.0)	− 3.5 (0.7)	− 1.2 (0.7)	1.7 (2.1)
Baseline UACR, mg/gCre, median (IQR)	20.0 (9.5–74.0)	29.7 (10.6–123.0)	20.0 (9.9–82.3)	20.0 (9.2–69.3)	18.2 (8.6–50.0)
Use of RAAS inhibitor, n (%)	4656 (37%)	452 (36%)	1052 (37%)	2111 (39%)	1041 (36%)
History of CVD, n (%)	1262 (10%)	127 (10%)	317 (11%)	561 (10%)	257 (8.9%)

*CKD* chronic kidney disease, *CVD* cardiovascular disease, *eGFR* estimated glomerular filtration rate, *IQR* interquartile range, *RAAS*, renin–angiotensin–aldosterone system,* SD* standard deviation, *UACR* urine albumin-to-creatinine ratio

### Association between the eGFR slopes and ESKD risk

**Fig. 2:**
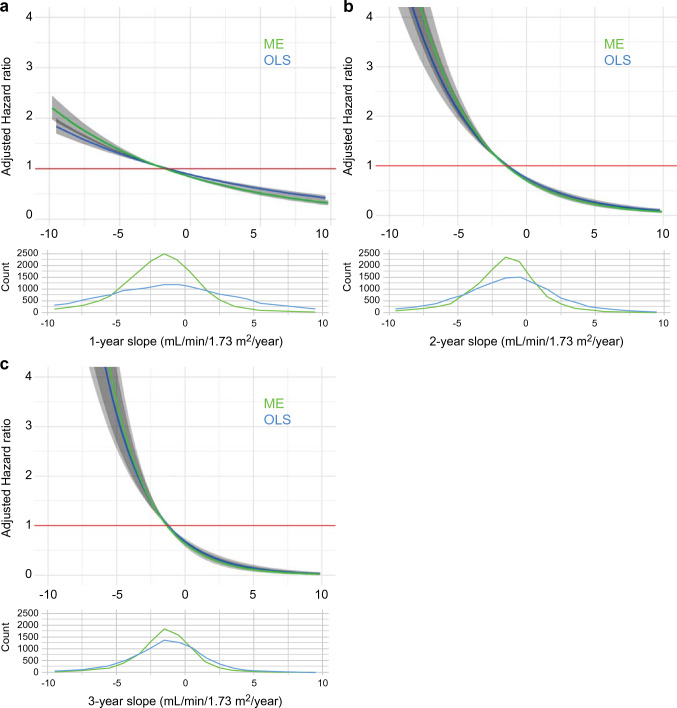
Population distribution of change in estimated glomerular filtration rate (eGFR) slope and the association between eGFR slope and composite end-stage kidney disease (ESKD) events. The upper panel shows the spline curve of the association between composite ESKD events and the (**A**) 1-year, (**B**) 2-year, and (**C**) 3-year eGFR slopes. The lower panel shows the distribution of the number of cases in whom (**A**) 1-year, (**B**) 2-year, and (**C**) 3-year eGFR slopes were calculated. The green line corresponds to the slope calculated under the mixed-effects model, and the blue line corresponds to the slope calculated under ordinary least-squares methods. To plot the spline curve, the average value of the eGFR slope was used as a reference, and the number of knots was set to three

Figure 2 shows the relationships between the 1-, 2-, and 3-year slopes calculated using ME model and OLS model and the risk of subsequent composite ESKD events. Whether the slope was calculated over 1, 2, or 3 years, the ME model and the OLS model showed the same trend, and the ME model correlated more strongly with the outcome. Even for the 1-year slope, the hazard ratio (HR) increased as the eGFR slope became steeper in the negative direction; this relationship became clearer as the slope calculation period increased.

**Fig. 3:**
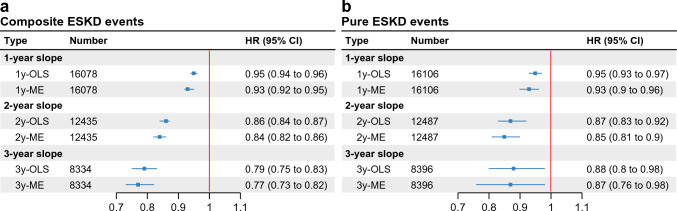
Association between end-stage kidney disease risk and estimated glomerular filtration rate (eGFR) slope calculated under mixed-effects (ME) model or ordinary least-squares linear regression (OLS) model. (**A**) Composite ESKD risk or (**B**) pure ESKD risk associations with the 1-year, 2-year, and 3-year eGFR slope reduction of 0.75 mL/min/1.73 m^2^/year, calculated under mixed-effects (ME) model or ordinary least-squares linear regression (OLS) model. *CI* confidence interval; *HR* hazard ratio

The HR was only 0.93 (95% confidence interval [CI] 0.92–0.95) for a reduction of 0.75 mL/min/1.73 m^2^/year in the 1-year eGFR slope calculated with the ME model, whereas it was 0.84 (95% CI 0.82–0.86) and 0.77 (95% CI 0.73–0.82) in the 2- and 3-year slopes, respectively (Fig. 3A).

A steeper eGFR decline was associated with higher risk of subsequent pure ESKD events, which was similar to the analysis for composite ESKD events (**Supplementary Fig. 1**). The hazard ratios for a reduction of 0.75 mL/min/1.73 m^2^/year in analyzing composite ESKD events were almost the same with those in analyzing pure ESKD events, particularly when the slope was calculated from a 1- or 2-year calculation period (Fig. 3A,B). However, for the 3-year slope, the HR for composite ESKD events and that for pure ESKD events were different because the observation period was short, and very few pure ESKD events occurred during that period (composite ESKD events N = 32, pure ESKD events N = 14).

### Subgroup analysis classified by baseline eGFR category

We divided the patients into the following two groups: the patients with preserved kidney function, who are in stages G1 or G2 (eGFR ≥ 60 mL/min/1.73 m^2^), and the patients with diabetes-induced relatively late-stage CKD, that are in stages G3a or G3b (eGFR 30–60 mL/min/1.73 m^2^). In the patients whose stages were G1-2, the 1-, 2-, and 3-year slopes were calculated for 11,337, 8785, and 5898 patients, with composite ESKD events occurring in 17 (0.15%), 15 (0.17%), and 5 (0.085%) patients, respectively. Note that this subgroup in G1-2 stages included approximately 65% of patients (1-year slope, 63.5%; 2-year slope, 65.4%; 3-year slope, 66.8%) with normal UACR who did not meet the definition of CKD, which resulted in a low incidence of ESKD events. In the patients in G3a-G3b stages, the 1-, 2-, and 3-year slopes were calculated for 4631, 3650, and 2436 patients, with composite ESKD events occurring in 62 (1.3%), 68 (1.9%), and 27 (1.1%) patients, respectively.

**Fig. 4:**
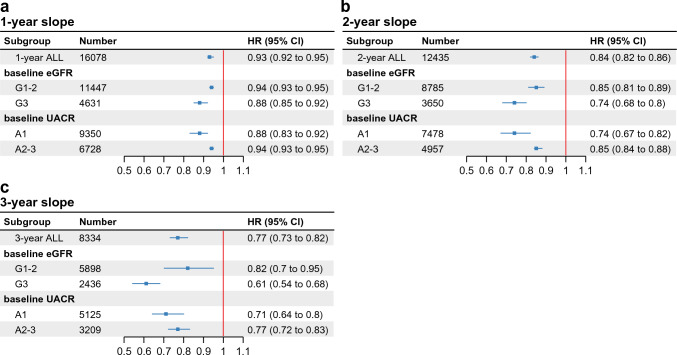
Association between composite end-stage kidney disease (ESKD) risk and estimated glomerular filtration rate (eGFR) slope: subgroup analysis. Composite ESKD risk associations with eGFR slope reduction of 0.75 mL/min/1.73 m^2^/year calculated under a mixed-effects model. eGFR slope was calculated over a period of (**A**) 1 year, (**B**) 2 years, and (**C**) 3 years, respectively. HRs and 95% CIs for each subgroup divided by eGFR (G1-2: ≥ 60 mL/min/1.73 m^2^, G3: 30–60 mL/min/1.73 m^2^), or urine albumin-creatinine ratio (A1: < 30 mg/gCre, A2-3, ≥ 30 mg/gCre) were shown. *CI* confidence interval, *HR* hazard ratio.

As in the main analysis, the HR increased as the eGFR slope became steeper in the negative direction both in the G1-2 group and the G3a-G3b group (**Supplementary Fig. 2**). This relationship was more evident in the group of G3a-G3b stages than that of G1-2 stages. It was stronger the longer the eGFR slope calculation period was (Fig. 4A-C).

For a 2-year slope reduction of 0.75 mL/min/1.73 m^2^/year, the HR was 0.85 (95% CI 0.81–0.89) and 0.74 (95% CI 0.68–0.80) in the G1-2 group and the G3a-G3b group, respectively (Fig. 4B). The difference in the effect of slope reduction (the difference in aHR) between the two groups increased as the slope calculation period became longer (Fig. 4A-C, 1-year slope: 0.06; 2-year slope: 0.11; 3-year slope: 0.21).

### Subgroup analysis classified by baseline UACR category

Furthermore, we divided the patients into two groups according to the UACR (Note that the UACRs include the values complemented by multiple imputations). One group contains the patients with negative albuminuria, who are in stage A1 (UACR < 30 mg/gCre), and the other group contains the patients with positive albuminuria, that are in stages A2 or A3 (UACR ≥ 30 mg/gCre). In the patients whose stage was A1, the 1-, 2-, and 3-year slopes were calculated for 9350, 7478, and 5125 patients, with composite ESKD events occurring in 17 (0.18%), 18 (0.24%), and 11 (0.21%) patients, respectively. In this group with A1 stage, 77.4%, 77.0%, and 76.6% of the patients for whom 1-, 2-, and 3-year slope could be calculated had preserved kidney function (baseline eGFR ≥ 60 mL/min/1.73 m^2^). In the patients in A2 or A3 stages, the 1-, 2-, and 3-year slopes were calculated for 6728, 4957, and 3209 patients, with composite ESKD events occurring in 62 (0.92%), 65 (1.3%), and 21 (0.65%) patients, respectively.

In both subgroups, the HR increased as the eGFR slope became steeper in the negative direction (**Supplementary Fig. 3**). The association between slope and composite ESKD outcome was stronger in the A1 group than in the A2-3 group (Fig. 4A-C), regardless of whether the slope was calculated over 1, 2, or 3 years. The association was stronger in both A1 and A2-3 groups as the slope calculation period increased (Fig. 4A-C).

## Discussion

This study evaluated whether the overseas workshop recommendation [10] using the eGFR slope as a surrogate endpoint in early-stage CKD could be applied to the Japanese population. We analyzed the association between ESKD (the true endpoint) and eGFR slope (the surrogate endpoint) in J-DREAMS, an epidemiological database of Japanese patients with diabetes. We confirmed the eGFR slope as a surrogate endpoint of ESKD.

Analyses in overseas workshops [10] included meta-analyses of epidemiological cohorts, clinical trial data, and simulation data. Epidemiological cohort data was from 14 cohorts with approximately 3.9 million participants. Among these, 21% with a baseline eGFR ≥ 60 mL/min/1.73 m^2^ had DKD, while 28% with baseline eGFR < 60 mL/min/1.73 m^2^ had DKD. However, in this study, all patients had diabetes mellitus. 70.6% and 29.4% of patients with a 2-year slope had baseline eGFR values of ≥ 60 and < 60 mL/min/1.73 m^2^, respectively. Furthermore, 60.0% and 40.0% of the patients had normal (< 30 mg/gCre) and abnormal (≥ 30 mg/gCre) UACR values (including values complemented by multiple imputations). Only 53% of the patients (54.8%, 53.8%, and 52.7% of those with 1-, 2-, and 3-year slope calculability, respectively) had decreased eGFR and/or increased UACR values and were considered to have DKD. The above meta-analysis included two DKD-only cohorts, in which the HRs for an eGFR slope reduction of 0.75 mL/min/1.73 m^2^/year were similar to those in the overall meta-analysis. This indicates that the DKD cohort results may not differ significantly from the overall CKD results. The CKD meta-analysis results were similar to those of our study. In the previous meta-analysis, the HRs for a 2-year slope reduction of 0.75 mL/min/1.73 m^2^/year were 0.71 and 0.70 in patients with baseline eGFR values of < 60 and ≥ 60 mL/min/1.73 m^2^, respectively, when the slope was calculated under the mixed-effects model. In our analysis, the HRs were 0.74 and 0.85 in patients with baseline eGFR values of < 60 and ≥ 60 mL/min/1.73 m^2^ under the same conditions.

Patients with baseline eGFR values of ≥ 30 mL/min/1.73 m^2^ (CKD stage G1-G3b), known as “early-stage CKD” with diabetes, were analyzed. The HRs were higher when the slope was steeper in the negative direction and lower when it was steeper in the positive direction (Fig. 2). The HRs for the eGFR slope reductions of 0.75 mL/min/1.73 m^2^/year and their 95% CIs were < 1, regardless of the slope used (Fig. 4A-C)**.** Despite specific HR values disparities, the results of our study were consistent with those of the epidemiological cohort meta-analyses in the workshop mentioned above [10, 13].

The association between the eGFR slope and ESKD risk became stronger as the calculation period lasted longer. In the sensitivity analysis, using a listwise method without multiple imputations (**Supplementary Table 1**), the 1-year and 2-year slopes HRs were similar to those in the main analysis. However, the HR for the 3-year slope differed. There was no trend toward a stronger association as the slope calculation increased. The large percentage of missing UACR data, especially in the 3-year slope (1-year slope: 56.6%, 2-year slope: 60.6%, and 3-year slope: 67.2%), a large difference in the number of cases used in the analysis, and the fact that the longer the slope calculation period, the fewer the number of events may explain this discrepancy. Events during the slope calculation period were excluded from the analysis.

We examined the relationship between composite ESKD events and eGFR slope calculated using the ME model or OLS model, respectively (Fig. 2A-C, 3A). The overseas workshop reported that the eGFR slope calculated using the ME model correlated more significantly with ESKD than that calculated using OLS [10]. Our results were consistent with this finding.

Because the observation period was short, we focused on the association between eGFR slope and composite ESKD events, which includes the start of kidney replacement therapy and records of eGFR < 15 mL/min/1.73 m^2^. We also found similar HRs for pure ESKD events and eGFR slope, especially for 1-year and 2-year slope (Figs. 2,3, **Supplementary Fig. 1**).

Since the subsequent eGFR slope was expected to be affected by the baseline eGFR, we also analyzed groups based on CKD G stage: stages G1–G2 and stages G3a–G3b. The HR increased in both groups as the eGFR slope became steeper in the negative direction and decreased as the eGFR slope became steeper in the positive direction (**Supplementary Fig. 2**). The relationship between the ESKD (true endpoint) and eGFR slope (surrogate endpoint) was stronger in the group with more advanced CKD (G3a-G3b) than in the group with early CKD. The association between slower eGFR decline rate and lower future ESKD risk is sustained in patients with baseline eGFRs of > 60 ml/min/1.73 m^2^; however, the greatest benefit is expected in patients with the most risk of future ESKD [13]. Thus, our results suggested that it may be better to examine the association between eGFR slope and ESKD independently for CKD G1-2 and G3.

Another subgroup analysis was performed based on CKD A stage: stage A1 and stages A2-3. The albuminuria negative group (A1 stage) had a stronger correlation between eGFR slope and composite ESKD events than the albuminuria positive group (A2-3 stages), suggesting that eGFR slope may be particularly useful in the albuminuria negative group (Fig. 4, **Supplementary Fig. 3**). However, patients with missing baseline UACR values were classified by multiple imputation. Analysis of large data sets that do not require multiple imputations is a subject for future research.

This study had some limitations. First, this was an epidemiological data-based observational study; interventional studies may have yielded different results. Second, death in the database was optional and needed to be verified; thus, we could not analyze the eGFR slope and death risk. Third, the high percentage of missing UACRs may have affected the results despite multiple imputations. Fourth, the average observation period for events in this study was approximately 1 year, and further ESKD events were not evaluated.

## Conclusion

J-DREAMS, a representative database of Japanese patients with diabetes, was used to examine the relationship between eGFR slope and ESKD. Furthermore, we examined whether the association between a slower eGFR slope and a lower risk of ESKD occurrence, presented at an overseas workshop, was similar in Japanese patients with early-stage DKD. Our study results suggest that changes in the eGFR slope may increase the risk of ESKD in Japanese patients with diabetes.

### Supplementary Information

Below is the link to the electronic supplementary material.

Supplementary file1 (TIF 1121 KB)

Supplementary file2 (TIF 1003 KB)

Supplementary file3 (TIF 767 KB)

Supplementary file4 (DOCX 17 KB)
