# Supplementary figures and images for: eGFR slope as a surrogate endpoint for end-stage kidney disease in patients with diabetes and eGFR > 30 mL/min/1.73 m2 in the J-DREAMS cohort
Source: Clin Exp Nephrol. 2023 Oct 9;28(2):144–52. doi: 10.1007/s10157-023-02408-z (PMC10808312; doi:10.1007/s10157-023-02408-z)

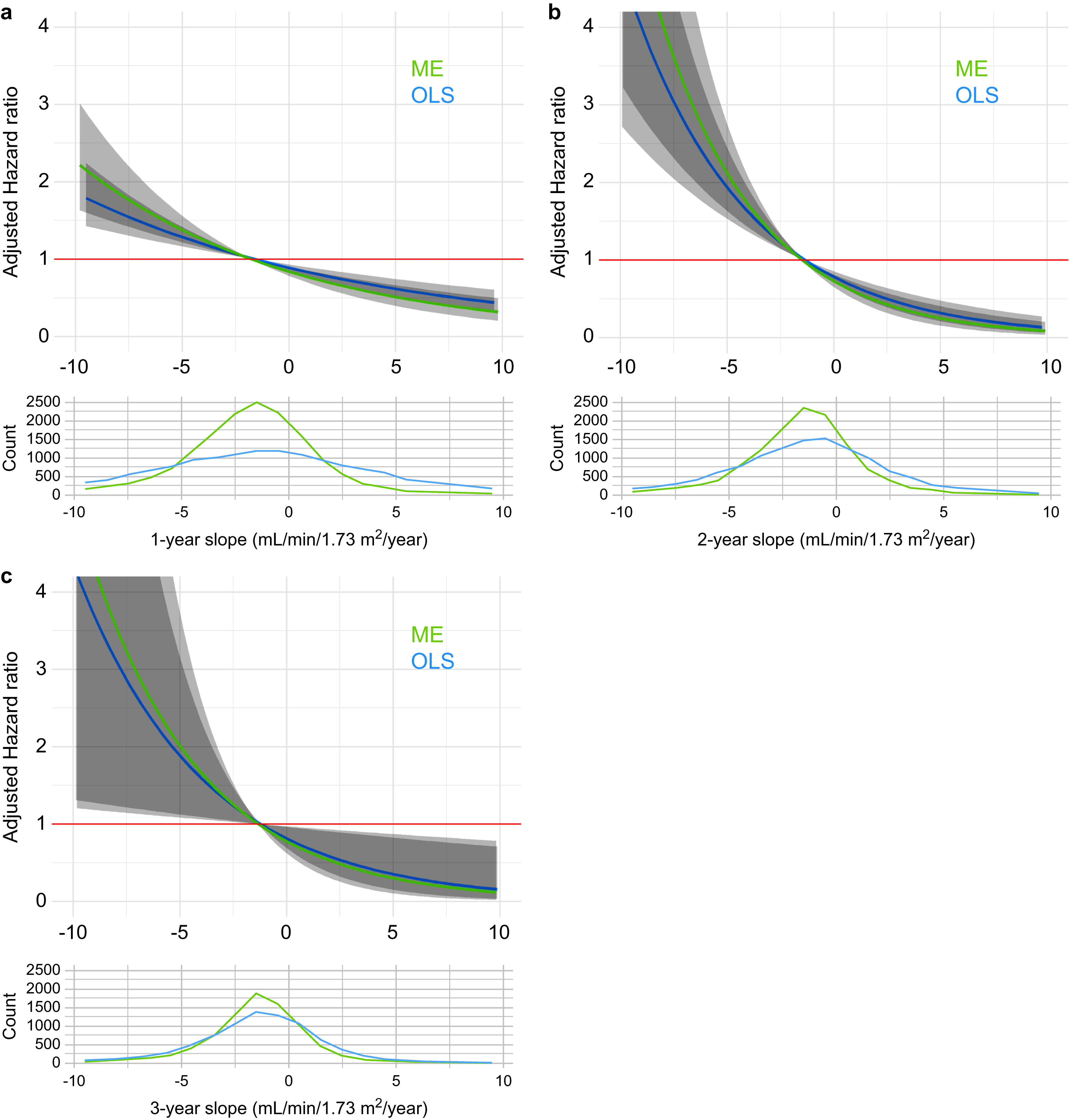

Supplement: Supplementary file 1 — Supplementary file1 (TIF 1121 KB) [file 10157_2023_2408_MOESM1_ESM.tif]

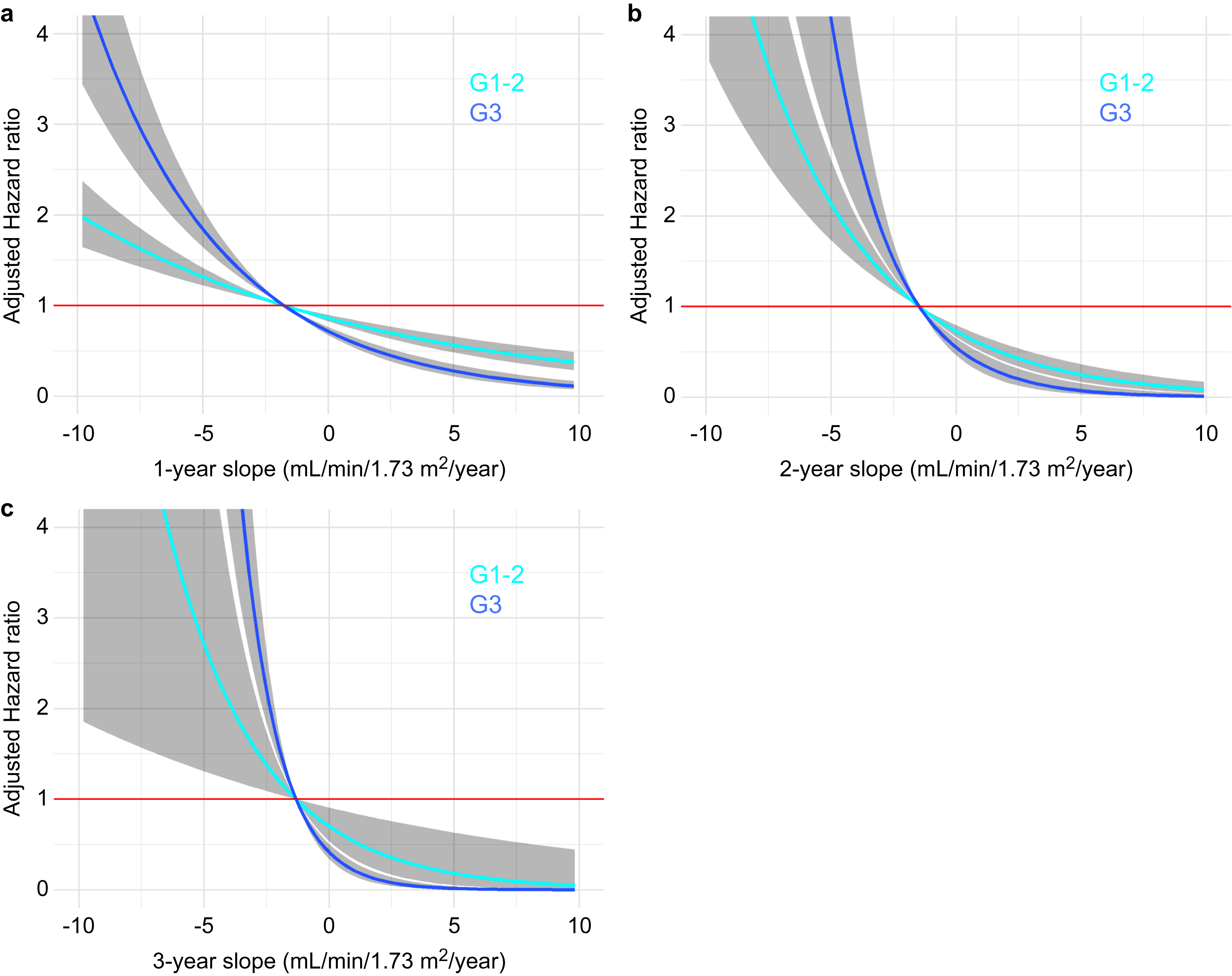

Supplement: Supplementary file 2 — Supplementary file2 (TIF 1003 KB) [file 10157_2023_2408_MOESM2_ESM.tif]

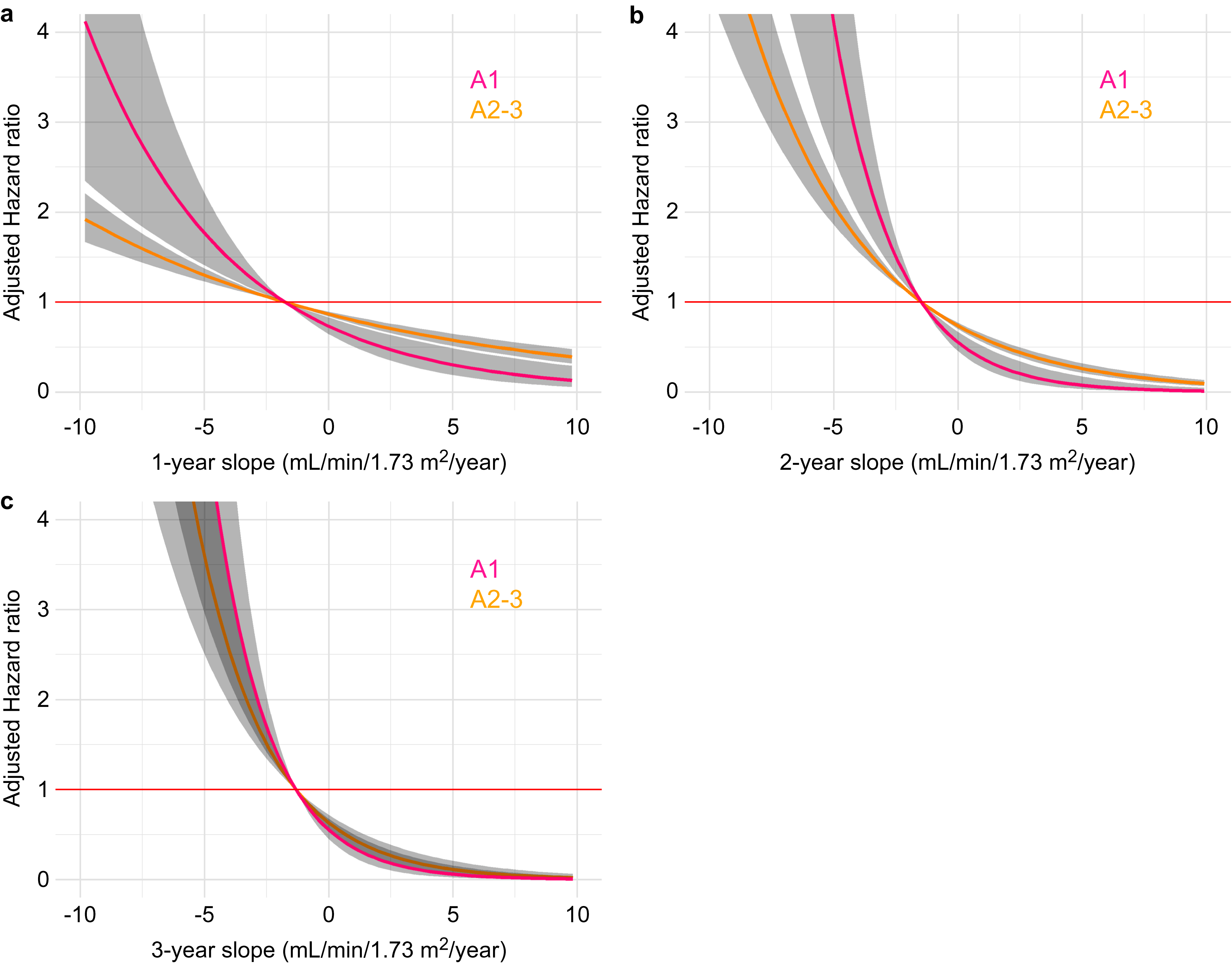

Supplement: Supplementary file 3 — Supplementary file3 (TIF 767 KB) [file 10157_2023_2408_MOESM3_ESM.tif]
