# Supplementary material for: eGFR slope as a surrogate endpoint for end-stage kidney disease in patients with diabetes and eGFR > 30 mL/min/1.73 m2 in the J-DREAMS cohort
Source: Clin Exp Nephrol. 2023 Oct 9;28(2):144–52. doi: 10.1007/s10157-023-02408-z (PMC10808312; doi:10.1007/s10157-023-02408-z)
Supplement: Supplementary file 4 — Supplementary file4 (DOCX 17 KB) [file 10157_2023_2408_MOESM4_ESM.docx]

**Supplementary table 1. Association between composite end-stage kidney disease risk and 0.75 mL/min/1.73 m^2^/year eGFR slope reduction when analyzed by listwise method as a sensitivity analysis**

|  | N | HR (95% CI) |
| --- | --- | --- |
| 1-year slope | 6,983 | 0.94 (0.91-0.97) |
| 2-year slope | 4,902 | 0.85 (0.79-0.91) |
| 3-year slope | 2,736 | 0.90 (0.63-1.28) |

HR: hazard ratio, CI: confidence interval.
